# Inequalities and Determinants of Quality Antenatal Care Coverage Among Women of Reproductive Age (15–49) in Tanzania: A Cross‐Sectional Analysis of National Data

**DOI:** 10.1002/hsr2.71081

**Published:** 2025-07-16

**Authors:** Sanun Ally Kessy, Sophia A. Kagoye, Jovin R. Tibenderana, Ahmed Yusuph Nyaki, Rachel N. Manongi, Innocent B. Mboya

**Affiliations:** ^1^ Directorate of Training and Research, Benjamin Mkapa Hospital Dodoma Tanzania; ^2^ Ifakara Health Institute Dar es Salaam Tanzania; ^3^ National Institute for Medical Research Mwanza Tanzania; ^4^ Department of Public Health St. Francis University College of Health and Allied Sciences Ifakara Tanzania; ^5^ Department of Epidemiology and Biostatistics, School of Public Health KCMC University Moshi Tanzania; ^6^ Africa Academy for Public Health Dar es Salaam Tanzania

**Keywords:** antenatal care, inequalities, quality antenatal care coverage, Tanzania

## Abstract

**Background and Aims:**

Maternal and neonatal mortality remain high in sub‐Saharan Africa (SSA), which can be reduced by the expanded antenatal care (ANC) services intervention. Challenges exist in the quality coverage of ANC in Tanzania. We aimed to assess inequalities and determinants of quality ANC coverage among women of reproductive age (WRA, 15–49 years) in Tanzania.

**Methods:**

We performed a secondary analysis of cross‐sectional data from 6620 women of reproductive age using the 2022 Tanzania Demographic and Health Survey. Quality ANC coverage (ANCq) was measured using seven indicators, with scores ≥ 7 classified as adequate. Log‐linear regression models were used to determine factors associated with ANCq using Stata version 18.0. Inequalities in ANCq by age categories, education level, socioeconomic status (SES), area of residence, and geographical zones were analyzed using WHO HEAT 3.1 through Difference and Population Attributable Risk measures.

**Results:**

Among 6620 WRA (43%), had adequate ANCq coverage. Higher inequalities were observed among women with low education, low SES, rural residence, and living in western zones. ANCq coverage was positively associated with high SES (RR 1.35, 95% CI: 1.20–1.52), secondary/higher education (RR 1.21, 95% CI: 1.06–1.38), high media exposure (RR 1.11, 95% CI: 1.00–1.21), easy access to health facility (RR 1.11, 95% CI: 1.01–1.27), and partner's higher education (RR 1.28, 95% CI: 1.08–1.52). Inverse associations were observed among older women (35–49 years) (RR 0.89, 95% CI: 0.80–0.97) and those with higher birth order (RR 0.78, 95% CI: 0.72–0.84).

**Conclusion:**

The study demonstrates suboptimal ANCq coverage in Tanzania, especially among disadvantaged groups, which quality ANCq having a positive association with rich wealth status, higher education level, high media exposure, health facility access, and older maternal age. Addressing these socioeconomic disparities is crucial for improving ANCq coverage and maternal and child health outcomes.

**Abbreviations**
AIC: Akaike information criteria
ANC: antenatal care
BIC: Bayesian information criteria
CHF: community health fund
CI: confidence interval
DHS: demographic and health survey
EA: enumeration area
HI: health insurance
LMIC: low and middle income country
NBS: national bureau of statistics
NSSF: national social security fund
PR: prevalence risk
PSU: primary sampling unit
SDGs: sustainable development goals
TDHS: Tanzania demographic and health survey
UHC: universal health coverage
VIF: variance inflation factor
WRA: women of reproductive age

## 1 Background

Maternal and neonatal mortality remains high in sub‐Saharan Africa (SSA) [1]. In 2017, SSA and Asia together constituted 86% of maternal deaths worldwide, with SSA accounting for two‐thirds of these fatalities [2]. In addition, SSA has the highest neonatal mortality rate, accounting for 43% of newborn deaths globally [3]. To improve maternal and neonatal outcomes, quality antenatal care (ANC) provided by skilled professionals throughout pregnancy is essential [4]. Numerous studies conducted in low‐ and middle‐income countries (LMICs) indicate that improving the quality and equity of ANC has the potential to save 67 million neonatal lives [5], and women of reproductive age (WRA) who received quality ANC coverage experienced a 34% reduction in newborn mortality risk. This underscores the essential role of ANC in enhancing newborn health [6].

In Tanzania, there has been a significant drop in maternal deaths over 7 years from 540 deaths per 100,000 live births in 2015 to 104 deaths per 100,000 live births in 2022. Despite this progress, neonatal mortality has only decreased by 1% from 2016 to 2022 [7, 8]. This slow decrease brings challenges for healthcare and the economy and is an indicator of significant inequalities in the provision of maternal health services during pregnancy [5, 9, 10, 11], especially ensuring quality coverage of ANC interventions [12, 13]. Although in LMICs most WRA attend ANC in their most recent pregnancy, that is, crude coverage (60%–94%), challenges remain with the quality of ANC coverage, ranging between 13% and 63% [14]. Research indicates that poor care, not just lack of access, contributes to inequalities in ANC coverage [15]. Expanding the focus beyond access to care has led to increased interest in measuring quality coverage as critical to achieving universal health coverage [16]. Nevertheless, quality coverage offers a more comprehensive measurement, aligning healthcare needs, utilization, and service quality [17]. Much scholarly research globally and in Tanzania has focused on crude rather than quality coverage of ANC interventions [14, 18].

The study aimed to assess inequalities and factors associated with quality coverage of antenatal care interventions among women of reproductive age (15–49) in Tanzania.

## 2 Methods

### 2.1 Data Source

The study utilized secondary data from the 2022 Tanzania Demographic and Health Survey (TDHS). A detailed description of the DHS methodology is available elsewhere [7, 8]. Briefly, TDHS is a nationwide cross‐sectional survey done every 5 years since 1991–1992 and collects demographic and health information on marriage, fertility, family planning, reproductive health, and child health. The survey aims to aid policymakers and program managers in monitoring, evaluating, and designing programs and initiatives to improve the population health [8]. The study population in the TDHS included all women aged 15–49 years, all men aged 15–49 years, and all children under 5. The survey uses a multi‐stage sampling, where enumeration areas (EAs) from the 2022 Tanzanian population and housing census functioned as clusters, from which a systematic random sample of households was selected. Additionally, TDHS collects data using standardized questionnaires, separately for households, women, men, and biomarkers [7, 8].

### 2.2 Setting

The survey was conducted in Tanzania, East Africa's largest country, covering 940,000 km^2^, including 60,000 km^2^ of inland waterways. Tanzania is divided into 9 zones, encompassing 31 regions and 184 districts. The population is projected to reach 67.7 million by 2025, with an annual growth rate of 3.2% [8]. Additionally, the average fertility rate is 5.5 children per woman [8]. The number of health centers in Tanzania increased by 16.1% from 8449 in 2019 to 10,067 in 2022. To ensure the availability of essential medicines in these facilities, the government has invested 20 billion Tanzanian Shillings per month ($8,550,662).

### 2.3 Study Population

**Figure 1:**
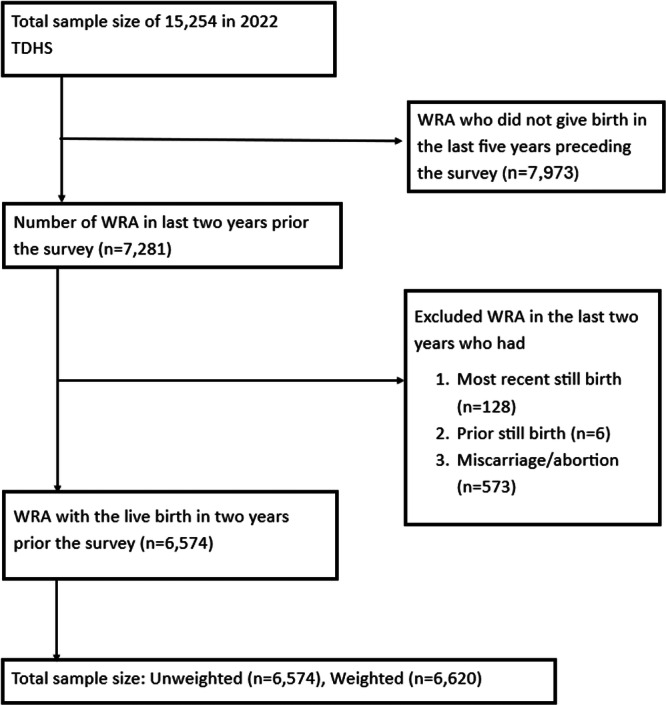
Flow chart for selection of study participants. Abbreviations: HH, household; TDHS, Tanzania demographic and health survey; WRA, women of reproductive age.

The study utilized data from the 2022 Tanzania Demographic and Health Survey (TDHS), starting with a total sample of 15,254 women of reproductive age (WRA). Women who did not give birth in the 5 years preceding the survey (*n *=7973) were excluded. Additionally, women who experienced adverse pregnancy outcomes in the last 2 years, including most recent stillbirths (*n *= 128), prior stillbirths (*n *= 6), or miscarriages/abortions (*n *= 573), were also excluded from the analysis. The final inclusion criteria focused on women who had their live birth within the 2 years preceding the survey. This narrowed the sample size to 6574 unweighted participants, with a weighted sample size of 6620 (Figure 1).

### 2.4 Measurements

The dependent variable was the crude and quality ANC coverage (ANCq). Crude ANC coverage referred to the proportion women who have given birth in the last 2 years and had attended at least one ANC visit with a skilled attendant for their last birth [8]. ANCq was defined as the proportion of women aged 15–49 years who came into contact with ANC services that are ready and received ANC according to the quality of ANC care standards [13]. Standard quality of ANC coverage includes the WHO‐stipulated components (laboratory tests, physical examination, and history taking), health promotion (pregnancy information, nutrition advice, subsequent contraception, birth planning, immunization, and breastfeeding), and care provision [1].

The ANCq content qualified indicator was used to measure quality coverage. ANCq is a novel survey‐based ANC indicator calculated as a score, composed of seven variables [19]. The ANCq score ranges from 0 to 10 points (0 indicating no ANC and higher scores indicating quality ANCq coverage), Table 1. ANCq was dichotomized into a binary variable whereby a score of ≥ 7 was classified as quality ANCq coverage (“Yes”), and “No” if otherwise [19, 20].

**Table 1:** Scoring of the variables that comprise the content‐qualified ANC coverage indicator (ANCq).

Contact with ANC services	Response	Score
Number of ANC visits	0 visits	0
	1–3 visits	1
	4–7 visits	2
	8 or more visits	3
ANC started in the first trimester	No	0
	Yes	2
ANC content^a^		
Skilled provider in at least one visit	No	0
	Yes	1
Blood pressure measured	No	0
	Yes	1
Blood sample collected	No	0
	Yes	1
Urine sample collected	No	0
	Yes	1
Received tetanus toxoid (at least two shots)	No	0
	Yes	1

a ANC content; antenatal care content (based on ANCq indicator measurement using five components.

The independent variables in this analysis included socio‐demographic and economic characteristics. Socio‐demographic characteristics included age in years and categories [3, 14, 15, 16, 17, 18, 19, 20, 21, 22, 23, 24, 25, 26, 27, 28, 29, 30, 31, 32, 33, 34, 35, 36, 37, 38, 39, 40, 41, 42], marital status (married/in union, unmarried—that included divorced, never in union, no longer living together/separated, living with partner, widowed), WRA and partner's highest education level (no education, primary, secondary or higher), birth order (1 child, ≥ 2 children), level of media exposure (high, low), distance to health facility (big problem, not big problem), and geographical zones. Socioeconomic characteristics include employment (employed, not employed) and household wealth tertiles (poor, middle, rich).

### 2.5 Statistical Analysis

Data analysis accounted for the complex nature of the DHS survey design by considering sampling weights, in which over‐represented observations are weighted down, and underrepresented observations are weighted up to reflect the target population [21]. EAs were considered as clusters while also accounting for rural versus urban stratification. Survey means with standard deviations (SD) summarized numeric variables, frequencies and percentages for categorical variables. The proportion of ANCq coverage was compared across participant characteristics using *χ* ^2^ test.

The WHO Health Equity Assessment Toolkit (HEAT) software version 3.1 [1] was used to assess ANCq coverage inequalities by wealth status, education level, area of residence, age categories, and geographical zones. The inequality in ANCq coverage was analyzed by disaggregating the ANCq coverage by equity stratifiers, followed by estimating two equity measures—“Difference” and Population Attributable Risk (PAR) [19]. The “Difference” was calculated by taking the highest minus the lowest proportion of quality ANCq between groups. To calculate PAR, we let *μ* represent the population average of ANCq coverage, and *P*_ref_ represent the proportion of ANCq coverage in the reference group of the variables of interest. The PAR is then obtained by subtracting *μ* from *P*_ref_. For the ordered variables (wealth status, age categories, and education level), the reference group was the most advantageous group, and for the unordered variables (geographical zones and residence), the reference group was the subgroup with highest prevalence. Statistically significant inequalities in ANCq coverage existed if the 95% confidence intervals (CI) for the Difference (*D*) and PAR did not include a null value of 0 [22].

The log‐linear regression (generalized linear model with the Poisson family and log‐link function) determined the factors associated with ANCq coverage. The model estimated the risk ratio/relative risk (RR) and 95% confidence interval (CI) for these associations. Independent variables with a *p* value of < 5% in bivariate analyses and those known to influence ANC coverage were further analyzed in the multivariable regression models. Manual stepwise regression was used to evaluate the confounding effect and contribution of these factors on ANCq coverage. A variable was a confounder if its inclusion in the adjusted analyses changed the risk ratio of other independent variables by at least 10%. The models with the lowest Akaike information criteria (AIC) were considered more parsimonious. Multicollinearity between exposures was checked using VIF (variance inflammation factor), with no evidence observed. All statistical tests were evaluated at a two‐tailed alpha level of 0.05. All statistical analyses were performed in STATA version 18.0 (StataCorp LLC, College Station, Texas, USA).

## 3 Results

### 3.1 Social‐Demographic Characteristics of the Respondents

Of the 6620 eligible WRA, the mean age (SD) was 29.19 (7.2) years, and more than one‐third (36.0%) were aged 15–24 years, while 42.9% were aged 25–34 years. Nearly three‐quarters (72.1%) resided in rural areas, and over half (55.4%) were married. In terms of education, over one‐quarter (27.9%) had secondary or higher education, while 52.6% had primary education. Two‐thirds (67.6%) were employed, and 44.5% had a birth order of two or more children. Regarding household wealth, 36.4% were from rich households. Approximately one‐third (31.9%) reported distance to a health facility as a big problem (Table 2).

**Table 2:** Distribution of respondents' social‐demographic characteristics in the 2022 TDHS (*N* = 6620).

Variables	Frequency	Weighted %	95% CI
Age (years)			
15–24	2385	36.0	(34.6, 37.4)
25–34	2842	42.9	(41.4, 44.4)
35–49	1393	21.1	(20.0, 22.2)
Mean (SD)	29.19 (7.2)		
Educational level			
No education	1287	19.4	(18.1, 20.7)
Primary	3486	52.6	(51.0, 54.3)
Secondary/higher	1847	27.9	(26.3, 29.4)
Wealth status			
Poor	2105	31.8	(30.4, 33.2)
Middle	2107	31.8	(30.2, 33.3)
Rich	2408	36.4	(34.7, 38.0)
Birth order			
One child	3678	55.5	(53.8, 57.2)
Two or more children	2942	44.5	(42.8, 46.2)
Marital status			
Unmarried^a^	2951	44.6	(43.1, 46.2)
Married	3669	55.4	(53.8, 56.9)
Employment status			
Not employed	2142	32.4	(31.0, 33.8)
Employed	4478	67.6	(66.2, 69.0)
Partner's education			
No education	726	11.0	(9.9, 12.2)
Primary education	4178	63.1	(61.5, 64.7)
Secondary/higher	1716	25.9	(24.4, 27.4)
Residence			
Urban	1848	27.9	(26.3, 29.5)
Rural	4772	72.1	(70.5, 73.7)
Distance to health facility^b^			
Big problem	2111	31.9	(30.4, 33.4)
Not a big problem	4509	68.1	(66.6, 69.6)
Geographical zones			
Zanzibar	179	2.7	(2.3, 3.1)
Western	646	9.8	(8.8, 10.9)
Northern	696	10.5	(9.4, 11.6)
Central	635	9.6	(8.5, 10.7)
Southern highlands	367	5.5	(4.8, 6.2)
Southern	292	4.4	(3.7, 5.1)
Southwest highlands	620	9.4	(8.3, 10.5)
Lake	2036	30.7	(29.1, 32.3)
Eastern	1149	17.4	(15.9, 18.9)
Level of media exposure			
Low	3297	49.8	(48.2, 51.4)
High	3323	50.2	(48.6, 51.8)

*Note:* All percentage and sample size are weighted.

a Unmarried—that included divorced, never in union, no longer living together/separated, living with partner, widowed.

b Distance to health facility. Women were asked about their perception on distance; no cutoff point was used.

### 3.2 Crude and ANCq Coverage of ANC Interventions in Tanzania 2022

**Figure 2:**
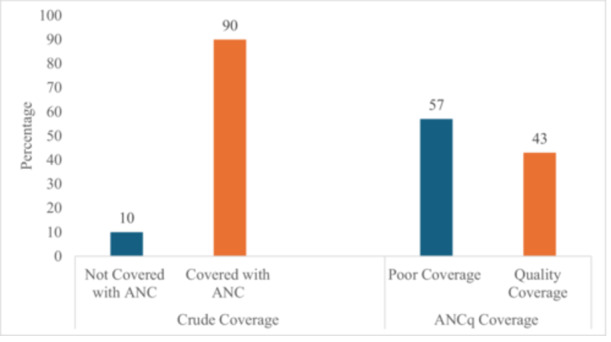
Distribution of the crude and ANCq coverage of ANC Interventions in Tanzania 2022. All frequencies and percentages are weighted. ANCq coverage constituted individuals scoring ≥ 7 on the seven indicators related to ANC contact and content of care received in the most recent pregnancy. Abbreviations: ANC, antenatal care; ANCq, antenatal care content‐qualified coverage.

While 90% of WRA in Tanzania have attended ANC in their most recent pregnancy, only 43% reported ANCq coverage (Figure 2).

### 3.3 Inequality in ANCq Coverage Among WRA in Tanzania

Findings show significant inequalities in ANCq coverage by age, education level, socioeconomic status (SES), area of residence, and geographical zones. ANCq coverage was highest among women in the richest wealth quintile (44%) compared to the poorest (17%), and among those with secondary or higher education (29%) compared to those with no education or only primary education (17%). Similarly, urban residents (45%) had markedly higher ANCq coverage than their rural counterparts (24%). Subnational disparities were also observed: women in the Southern (47%), Southern Highlands (41%), and Eastern (41%) zones had substantially higher ANCq coverage compared to those in other regions, such as Western (20%) and Lake (21%) (Table 3).

**Table 3:** Inequality in ANCq coverage among WRA in Tanzania.

Variable	Number of individuals	Number with ANCq	Proportion (%)	95% CI
Age (years)				
15–24	2385	738	30.9	28.7, 33.1
25–34	2842	841	29.6	27.5, 31.6
35–49	1393	308	22.1	19.6, 24.6
Educational level				
No education/primary	4774	801	16.8	14.7, 18.8
Secondary/higher	1846	530	28.7	26.5, 31.0
Wealth status				
Poorest	1328	228	17.2	15.1, 19.3
Poor	1261	282	22.4	19.9, 24.8
Middle	1239	297	24.0	21.6, 26.4
Rich	1303	416	31.9	29.1, 34.8
Richest	1239	539	43.5	40.3, 46.7
Residence				
Rural	4772	1123	23.5	22.2, 24.9
Urban	1848	837	45.3	42.8, 47.9
Tanzania zones				
Zanzibar	179	55	30.7	25.0, 36.3
Western	646	128	19.8	16.3, 23.3
Northern	696	180	25.9	21.8, 29.9
Central	635	160	25.2	21.1, 29.3
Southern highlands	367	151	41.1	36.0, 46.2
Southern	292	137	46.9	41.4, 52.5
Southwest highlands	620	149	24.0	20.2, 27.9
Lake	2036	431	21.2	19.3, 23.1
Eastern	1149	465	40.5	36.7, 44.4

*Note:* All frequencies and percentages are weighted. ANCq coverage constituted individuals scoring ≥ 7 on the seven indicators related to ANC contact and content of care received in the most recent pregnancy.

Abbreviations: ANC, antenatal care; ANCq, antenatal care content‐qualified coverage; CI, confidence interval.

Further analysis using Difference and PAR inequality measures is shown in Table 4. The Difference measure of (28.6%, 95% CI: 28.6–28.7) and the PAR measure of 68.2% (95% CI: 68.1–68.3%) revealed substantial disparities, which were higher among richer subpopulations. The PAR measure of (10.6% 95% CI: 10.5–10.7) showed significant disparities, with lower coverage among non‐ and primary‐educated subgroups compared to those with secondary or higher education. Women with secondary or higher education had 9.1% percentage points more coverage than those without formal education.

**Table 4:** The percentage distribution of difference and PAR measures of inequalities in ANCq coverage by selected characteristics among WRA in Tanzania.

Variable	Difference (95% CI)	Population attributable risk (95% CI)
Wealth status (rich vs. poor)	28.6 (28.5–28.7)	68.2 (68.1–68.3)
Education level (secondary/higher vs. no education/primary)	9.0 (9.1–9.2)	10.6 (10.5–10.7)
Residence (urban vs. rural)	15.4 (15.3–15.5)	41.4 (41.5–41.7)
Geographical zones (southern vs. western)	10.0 (9.9–10.1)	8.8 (8.5–9.0)

*Note:* All frequencies and percentages are weighted. ANCq coverage constituted individuals scoring ≥ 7 on the seven indicators related to ANC contact and content of care received in the most recent pregnancy.

Abbreviations: ANC, antenatal care; ANCq, antenatal care content‐qualified coverage.

Also, there were urban–rural disparities in ANCq service coverage with PAR of 41.4% (95% CI: 41.5–41.7%). Urban women had 15.4% percentage points more coverage than those from rural areas. Furthermore, women in the Southern zone had 10% percentage points higher ANCq coverage than those from the Western zone (PAR = 8.8%, 95% CI: 8.5–9.0).

### 3.4 Factors Associated With ANCq Coverage Among WRA in Tanzania

In the crude analysis, ANCq coverage was associated with age, WRA, and partner's education level, area of residence, wealth status, birth order, employment status, distance to health facility, geographical zones, and level of media exposure (Table 5). However, in the multivariable regression analyses, only age categories (years), WRA and partner's education level, wealth status, birth order, employment status, area of residence, distance to health facility, and geographical zones remained as significant factors associated with ANCq coverage. A positive association (higher adjusted RR) with ANCq coverage was observed among WRA in the rich wealth status (RR 1.35, 95% CI: 1.20–1.52), with secondary/higher education (RR 1.21, 95% CI: 1.06–1.38), with high level of media exposure (RR 1.11, 95% CI: 1.00–1.21), those with no big problem with distance to health facility (RR 1.11, 95% CI: 1.01–1.27), and among WRA whose partners had secondary/higher education (RR 1.28, 95% CI: 1.08–1.52). An inverse association (lower adjusted RR) was observed among WRA aged 35–49 years (RR 0.89, 95% CI: 0.80–0.97), with birth order of ≥ 2 children (RR 0.78, 95% CI: 0.72–0.84) (Table 5).

**Table 5:** Factors associated with ANCq coverage among WRA 15–49 in Tanzania.

Variables	CRR (95% CI)	*p* value	ARR (95% CI)	*p* value
Age (years)				
25–34	1.00		1.00	
15–24	0.98 (0.91–1.07)	0.757	1.02 (0.94–1.10)	0.646
35–49	0.84 (0.76–0.93)	0.001	0.89 (0.81–0.98)	0.024
Educational level				
No education	1.00		1.00	
Primary	1.35 (1.20–1.52)	< 0.001	1.07 (0.95–1.20)	0.287
Secondary/higher	2.01 (1.79–2.26)	< 0.001	1.21 (1.06–1.38)	0.005
Wealth status				
Poor	1.00		1.00	
Middle	1.29 (1.16–1.43)	< 0.001	1.11 (0.99–1.23)	0.057
Rich	1.90 (1.75–2.06)	< 0.001	1.35 (1.20–1.52)	< 0.001
Birth order				
One child	1.00		1.00	
Two or more children	0.64 (0.59–0.69)	< 0.001	0.78 (0.72–0.84)	< 0.001
Marital status				
Unmarried	1.00			
Married	0.95 (0.89–1.02)	0.178	—	—
Employment status				
Not employed	1.00		1.00	
Employed	1.11 (1.03–1.21)	0.008	1.13 (1.04–1.22)	0.002
Partner's education				
No education	1.00		1.00	
Primary education	1.46 (1.25–1.69)	< 0.001	1.09 (0.93–1.27)	0.304
Secondary/higher	2.14 (1.83–2.50)	< 0.001	1.28 (1.08–1.52)	0.004
Residence				
Urban	1.00		1.00	
Rural	0.61 (0.57–0.66)	< 0.001	0.95 (0.86–1.05)	0.307
Distance to health facility				
Big problem	1.00		1.00	
Not a big problem	0.42 (1.31–1.55)	< 0.001	1.11 (1.01–1.27)	0.024
Tanzania zones				
Western	1.00		1.00	
Zanzibar	1.58 (1.34–1.86)	< 0.001	1.06 (0.89–1.25)	0.527
Northern	1.51 (1.27–1.80)	< 0.001	1.27 (1.06–1.51)	0.008
Central	1.42 (1.18–1.70)	< 0.001	1.28 (1.07–1.52)	0.006
Southern highlands	2.04 (1.73–2.39)	< 0.001	1.56 (1.32–1.83)	< 0.001
Southern	2.03 (1.69–2.43)	< 0.001	1.78 (1.47–2.11)	< 0.001
Southwest highlands	1.34 (1.13–1.59)	0.001	1.14 (0.96–1.34)	0.136
Lake	1.20 (1.01–1.42)	0.031	1.02 (0.86–1.20)	0.799
Eastern	2.12 (1.80–2.50)	< 0.001	1.46 (1.24–1.71)	< 0.001
Level of media exposure				
Low	1.00		1.00	
High	1.49 (1.39–1.60)	< 0.001	1.11 (1.00–1.21)	0.032

*Note:* All estimates (percentage and sample size) are weighted. ANCq, Antenatal Care content‐qualified coverage estimates were derived from a log‐linear regression model (Poisson family with log link function). The final multivariable model was adjusted for facility type and marital status.

Abbreviations: ANC, antenatal care; ARR, adjusted risk ratio; CI, confidence Interval; CR, crude risk ratio.

## 4 Discussion

The study assessed the inequalities and factors associated with quality coverage of ANC interventions among WRA in Tanzania. We found that quality ANCq coverage lagged considerably behind (43%) relative to the 90% crude ANC coverage. Higher ANCq coverage inequalities were among WRA with low education level, low SES, rural residents, and those from the Western Zones. Factors positively associated with ANCq coverage included WRA and partner secondary/higher education, rich SES, WRA who were employed, and having no problem with distance to health facility. Negative associations with ANCq were observed among older (35–49 years) WRA and those with a higher birth order of two or more children.

The notable gap between crude and ANCq coverage from our study has also been reported in other studies in SSA and Asia [23, 24]. A study in Ghana reported much higher quality ANCq coverage (70%) [25] than the current study, which was attributed to the early implementation of the free maternal health care policy in 2008, which provided women enrolled in the National Health Insurance Scheme (NHIS) with access to free maternal health services, including ANC [26]. The findings from our study indicate significant overestimation of the ANC coverage when only considering the crude coverage indicator, emphasizing the need for evaluating access and quality [3, 17, 27]. These findings also call for the government's focus on improving the quality of ANC services as a key health sector priority, especially within the maternal and neonatal health sector [3].

Significant disparities in quality of ANCq coverage were observed across WRA characteristics, with those in the advantaged groups (i.e., higher education level and higher SES) having higher coverage. This pattern persisted when investigating determinants of ANCq coverage, with higher likelihood observed among WRA from richer households and with secondary or higher education level, which aligns with previous research [28]. Educational initiatives play a vital role in augmenting health literacy among women, enabling them to prioritize maternal health and advocate for comprehensive ANC care [27], thus contributing significantly to improving maternal and child health outcomes. Also, education enhances women's decision‐making abilities and overall empowerment, better health literacy and economic status, enabling women to prioritize their health and promoting uptake of ANC services [29]. This positive trend extended to partners, whereby educated husbands are likely more supportive and involve women in the decision‐making process regarding ANC utilization, understand the importance of maternal healthcare, and possess better health literacy, enabling effective communication with healthcare providers [30].

ANCq coverage was higher among women residing in urban than rural areas and those residing in the eastern zones than the western zone. Previous studies in SSA have also reported higher ANCq coverage in urban than rural areas [31, 32]. This urban–rural inequality in ANCq is a significant concern with negative implications for maternal and child health. Building health facilities closer to urban populations enhances service use, even for mothers with lower SES. This approach reduces the barriers associated with distance and transportation costs, making it easier for urban women to access ANC services. Additionally, urbanization typically promotes greater utilization of services due to better infrastructure and accessibility [33]. However, in Tanzania, the government's efforts to increase the number of health centers and manpower in urban areas, alongside improving the budget for primary health care, appear to have positively impacted ANC coverage in these regions [34]. In contrast, women in Tanzania's rural areas confront substantial challenges that contribute to low ANCq coverage [35]. Because of the scarcity of health facilities and healthcare practitioners in rural areas, women are frequently forced to travel significant distances for care [36], which can be prohibitively expensive and time‐consuming. Rural locations also have poorer infrastructure, such as insufficient roads and transport choices, which further limit access to healthcare services [35]. Furthermore, rural communities have poorer socioeconomic statuses and lower levels of education, which have an impact on health literacy and maternal health service prioritization [37].

Proximity and travel time to health facilities impact health service utilization [38]. WRA without distance challenges were more likely to receive quality ANCq. A qualitative study in Zimbabwe found that long travel times exacerbated by poor road conditions hindered access to ANC services [30]. Several reasons explain this relationship. Long distances and travel times increase transportation costs, pose physical risks due to poor road conditions, and can conflict with work or childcare responsibilities. Additionally, distant facilities may be less integrated into the community, leading to lower awareness and trust in the services provided. These findings suggest that improving access to health services and ensuring the equitable distribution of healthcare facilities and personnel, particularly in remote and underserved areas, should be a priority to enhance ANC utilization and improve maternal and child health outcomes.

The findings of this study show that media exposure increased the ANCq coverage and was consistent with earlier research [29, 39]. A qualitative study in Tanzania revealed that women who have had media exposure are more aware than their peers about the benefits of ANC services during pregnancy and seek care from ANC providers that will help them live a healthy life [40]. The study also found that older mothers (< 35 years) and those with higher birth order (two or more children) were less likely to receive quality ANCq coverage. The finding is consistent with a previous study in Rwanda, where ANC utilization was lower in subsequent births [41]. Mothers with higher birth orders may perceive a lower need for ANC based on their previous pregnancy experiences while families with more children frequently experience higher financial and time constraints [42], restricting their ability to access and afford quality ANC services, Additionally, childcare obligations complicate ANC attendance, especially if facilities are inconvenient [39].

### 4.1 Strengths and Limitations of the Study

A strength of this study is the nationally representative sample of WRA in Tanzania, and an analysis of crude and quality ANC coverage and related inequalities. A limitation of this study is the lack of accounting for the variables known to influence ANCq coverage, such as availability of ANC services, self‐perceived health status, pregnancy‐related illness, and health facility and provider‐related factors [43, 44], which are not recorded in the TDHS. Additionally, the DHS cross‐sectional design does not allow establishing causality. Moreover, self‐reported ANC data introduces social desirability and recall bias, potentially leading to over‐ or underreporting of the utilization of ANC services.

## 5 Conclusion and Recommendation

Findings reveal significant gaps between crude (90%) and ANCq (43%) coverage. Quality ANCq was positively associated with WRA of rich wealth status, with secondary/higher education, high media exposure, no big problem concerning the distance to health facility, and partner's secondary/higher education. Inverse associations were observed among WRA aged 35–49 years and those with a high birth order of two or more children. Addressing disparities is crucial for better ANCq coverage and maternal and child outcomes. The Ministry of Health and local authorities need to establish mobile clinics in rural areas to provide ANC services and collaborate with NGOs to hold community workshops, using local leaders to promote early booking and regular visits.

## Author Contributions

**Sanun Ally Kessy:** conceptualization, data curation, formal analysis, methodology, visualization, writing – original draft, writing – review and editing. **Sophia A. Kagoye:** writing – original draft, writing – review and editing, visualization. **Jovin R. Tibenderana:** writing – original draft, writing – review and editing, visualization. **Ahmed Yusuph Nyaki:** writing – original draft, visualization, writing – review and editing, supervision. **Rachel N. Manongi:** writing – original draft, visualization, writing – review and editing, supervision. **Innocent B. Mboya:** writing – review and editing, writing – original draft, supervision, visualization.

## Ethics Statement

The 2022 TDHS adhered to all applicable national and international guidelines and regulations in its methodology. The protocol received review and approval from the National Institute for Medical Research. Additionally, permission from local authorities was secured. The authors obtained written permission from DHS to access the data set. Ethical approval to carry out the study was obtained from Kilimanjaro Christian Medical University College Research and Ethics Review Committee with reference number (# PG129/2023).

## Consent

Written informed consent was obtained from all participants and/or their legal guardians prior to data collection.

## Conflicts of Interest

The authors declare no conflicts of interest.

## Transparency Statement

The lead author, Sanun Ally Kessy, affirms that this manuscript is an honest, accurate, and transparent account of the study being reported; that no important aspects of the study have been omitted; and that any discrepancies from the study as planned (and, if relevant, registered) have been explained.

## Data Availability

The data set used is openly available upon permission from MEASURE DHS website (URL: https://www.dhsprogram.com/data/available-datasets.cfm).
